# Exploratory Investigation of Infrared Thermography for Measuring Gorilla Emotional Responses to Interactions with Familiar Humans

**DOI:** 10.3390/ani9090604

**Published:** 2019-08-25

**Authors:** Matthew R. Heintz, Grace Fuller, Stephanie Allard

**Affiliations:** Center for Zoo and Aquarium Animal Welfare and Ethics, Detroit Zoological Society, Royal Oak, MI 48067, USA

**Keywords:** animal welfare, human–animal interactions, infrared thermography, cortisol, oxytocin, positive reinforcement training, cognitive task, gorilla, emotion

## Abstract

**Simple Summary:**

Interactions between zoo professionals and animals, such as positive reinforcement training, occur regularly and are thought to be enriching for animals. However, there is little empirical information on how animals perceive these interactions or on the interactions’ effects on animals’ emotional states. Our objective was to assess the effectiveness of infrared thermography for measuring the emotional responses of three western lowland gorillas at the Detroit Zoo to routine interactions (positive reinforcement training and cognitive tasks) with familiar humans. In addition to thermal images, we collected saliva samples for hormone analysis before and after human–animal interactions and a control condition, and we recorded behavioral data during all conditions. Nasal temperatures consistently decreased for two gorillas during interactions, while the third gorilla showed repeated increases. The behavior of all three gorillas suggested that they were engaged in the interactions, without exhibiting behaviors that could indicate negative welfare impacts. Oxytocin and cortisol both decreased following all conditions, including the control, and were thus equivocal for interpreting the meaning of the changes in nasal temperature. As mixed results in previous research show, infrared thermography may detect emotional arousal; however, additional indicators are necessary to determine the valence of the observed changes. The variability in responses we observed do not lend themselves to making firm conclusions about the validity of infrared thermography (IRT) for measuring emotion in this context or about how these gorillas responded to interactions. Challenges and suggestions for future studies using infrared thermography to examine interactions between humans and zoo animals are discussed.

**Abstract:**

Interactions between zoo professionals and animals occur regularly and are believed to be enriching for animals. Little empirical information exists on how animals perceive these interactions, and particularly how the interactions affect the emotional states of animals. Infrared thermography (IRT) has shown some promise in the assessment of emotions in a variety of species, but further research is needed to determine if this method is useful in a zoo setting. We conducted a pilot study to determine if IRT is a valid measure of the emotional responses to routine interactions (positive reinforcement training and cognitive tasks, compared to a control condition) with familiar humans on three western lowland gorillas at the Detroit Zoo. We measured nasal temperatures associated with emotional change using IRT. To examine the validity of the IRT data, we collected saliva samples for hormone analysis before and after each condition, in addition to behavioral data during the interactions and control condition. Decreases in nasal temperatures for two gorillas and an increase in the third indicate that arousal changed consistently within individuals following the interactions but not the control condition. Pre-post cortisol levels and oxytocin concentrations decreased for all conditions, but the decreases seen did not differ among the conditions. The gorillas were highly engaged in the interactions, and two produced more grumble vocalizations during the human-animal interactions (HAIs) compared to the control condition. Additionally, the gorillas performed self-directed behaviors more often during the control condition, also suggesting HAIs were not a negative experience. In summary, we were able to measure changes in arousal using IRT, but we were unable to determine the emotional valence of these changes based on the additional indicators employed. Additionally, the inconsistency across these measures precluded firm conclusions about either the validity of IRT for measuring emotion in this context or how the interactions impacted these gorillas. These findings highlight the challenges of using IRT to measure emotional states in non-human animals, and we discuss further steps necessary to apply this method in future studies.

## 1. Introduction

The term human–animal interaction (HAI) refers to how animals in captivity act together with humans within their environment [1]. The quality and frequency of interactions can result in more complex and longer-term associations such as relationships and bonds, all of which have the potential to influence animal welfare either positively or negatively [2]. Although HAIs have been well researched in the agriculture [3] and companion animal fields [4], this is still a growing area of focus in the zoo and aquarium community [5]. Much of the existing research focuses on the impact of visitors on zoo animals; however, the ways in which animals perceive humans, including categorizing them as familiar versus unfamiliar, certainly plays a role in the effect of interactions. For example, western lowland gorillas (*Gorilla gorilla gorilla*) display more affiliative behaviors to familiar humans compared to unfamiliar humans [6]. There is a pressing need to better understand how interactions with familiar humans, namely animal care staff, may be perceived by animals and influence their welfare. Such interactions, including positive reinforcement training (PRT) and cognitive tasks, are thought to be stimulating for the animals and are therefore considered a type of environmental enrichment by promoting species-appropriate behaviors and reducing abnormal behaviors [7,8,9].

The welfare status of an individual animal encompasses its behavior, physiology and emotions. There are many proposed means to define “emotion” in nonhuman animals to guide scientific study (see Paul and Mendl [10] for a review). We follow the approach proposed by Anderson and Adolphs [11] that emotion represents an internal state triggered by stimuli (extrinsic or intrinsic) that causes observable behavioral, somatic and cognitive changes. Thus, to understand emotion—and by extension, welfare—in nonhuman animals, it is necessary to employ multiple measures and interpret them in concert. The success of this approach, however, depends on the utility of these measures. In particular, there is a dearth of behavioral and physiological measures that can be used to measure positive emotions [12]. As a result, many studies examining what are thought to be positive interactions between humans and nonhuman animals have relied on the reduction or absence of negative indicators to infer positive emotional responses. 

Examinations of the welfare impacts of interactions between animals and care or research staff have focused largely on behavioral responses. Unstructured and more formal PRT interactions between animal care staff and western lowland gorillas have been linked to decreases in abnormal and aggressive behaviors both for individuals [13] and in group settings [14]. In primates, self-directed behaviors (SDB) and other displacement behaviors occur in response to stressful situations and are therefore thought to reflect underlying anxiety or similar emotions [15]. In gorillas, interactions with animal care staff resulted in a decrease in abnormal and SDB but increased agonistic behavior [16]. Pomerantz and colleagues [17] demonstrated that PRT sessions with chimpanzees (*Pan troglodytes*) positively impacted their welfare through an increase in affiliative behaviors and a decrease in abnormal and stress-related behaviors. However, Herrelko and colleagues [18] found that chimpanzees demonstrated an increase in SDB during husbandry training compared to baseline. Black rhinoceros (*Diceros bicornis*), Sulawesi crested black macaques (*Macaca nigra*) and Chapman zebras (*Equus burchellii*) involved in a PRT program responded faster to cues from staff [19], suggesting that training sessions can also result in less fear of humans and potentially lead to improved relationships with animal care staff. Similar effects were seen in 17 species of new world primates, for which keeper-directed threats decreased after the establishment of a PRT program [20].

The impact of HAIs on physiological indicators of welfare has been investigated to a lesser degree. Studies have found that cortisol in dogs (*Canis familiaris*) either did not change [21,22] or increased [23] following human interaction. Explanations for the cortisol response in these situations include that the dogs may be anticipating play and preparing for activity [24], rather than experiencing stress from the interaction. Wolves (*Canis lupus*) and dogs had a decrease in salivary cortisol following training sessions [25], while several studies have found no immediate changes related to PRT (hamadryas baboons, *Papio hamadryas*, [26], bonobos (*Pan paniscus*) and Sumatran orangutans (*Pongo abelii*) [27]).

Oxytocin, a neuropeptide hormone associated with affiliation, social bonding and also involved in stress regulation [28], is starting to be used to assess the impact of interactions. This has been studied the most in dogs, and during positive interactions (e.g., grooming, gaze and verbal communication), oxytocin has been observed to increase in serum [22,23,29], urine [21,30] and saliva [29]. Although there has been variation in responses, including the influence of human sex differences [31] and another study that did not observe changes in oxytocin [32], the majority of studies demonstrate an increase in oxytocin following interactions with humans. The impact of HAIs on oxytocin has mainly been studied in domesticated species [33], which have been artificially selected to relate well to humans. Therefore, the relevance of these findings to non-domesticated species is unclear. To date, changes in oxytocin related to HAIs involving nonhuman primates have only been studied in a single group of western lowland gorillas. Leeds et al. [34] reported an increase in salivary oxytocin in a single gorilla following one unstructured play interaction with a primary keeper; however, Leeds [35] found no change in salivary oxytocin for two gorillas following PRT sessions. These results suggest oxytocin may have promise for studying HAIs in nonhuman primates, but more research is needed.

Infrared thermography (IRT) has increasingly been used in the study of emotions and has shown some promise as a non-invasive tool for animal welfare assessments. Changes in arousal can occur in response to activation of the sympathetic nervous system (SNS) and lead to changes in blood flow, resulting in temperature changes in different areas of the face (reviewed in [36]). These changes can be captured by an IRT camera [37]. Dogs experienced an increase in eye temperature during examinations performed by unfamiliar veterinarians [38]. Additionally, in horses (*Equus callabus*), eye temperature increased following exposure to a novel object meant to elicit a fear response [39]. In non-human primates, temperature decreases in the nasal region have most often been related to negative experiences such as threatening stimuli (rhesus macaques, *Macaca mulatta*, [40,41]) and exposure to audio and video recordings of conspecific fights (chimpanzees [42]). However, the effect of presumably positive experiences has also resulted in increases in nasal temperature, as seen in rhesus macaques following gentle sweeping of the back by a familiar experimenter [43]. Therefore, based on existing studies, it is not entirely clear whether to expect an increase or decrease in temperature associated with arousal, or whether the direction of change consistently indicates the valence of the underlying emotional response.

The goal of this study was to assess the reliability and validity of IRT as a measure of the emotional responses of gorillas to interactions with familiar humans. Conditions included participation in an established cognitive research protocol, a training session with a familiar animal care staff member and a control. We made the following assumptions. If IRT responses were consistent over time within individuals, it would indicate good reliability for this method. If responses differed between the interactions and control condition, it would indicate that IRT is indeed measuring responses to the interactions, suggesting good construct validity [44]. Finally, concordance of the IRT data with behavioral and endocrine responses to the interactions would further support the validity of IRT for measuring these responses [44]. We predicted that nasal temperatures would change, indicative of arousal, salivary cortisol would decrease and salivary oxytocin would increase following interactions. We also predicted that the animals would demonstrate positive behavioral responses to the interactions, as made evident by low levels of breaking away from the interactions and of SDB, as well as higher rates of positive vocalizations during HAIs than the control. Taken together, these indicators would suggest a positive emotional response to the interactions by the gorillas, and their consistent agreement with changes in nasal temperature would suggest IRT is a reliable and valid measure of emotional responses to these interactions.

## 2. Materials and Methods

This study was approved by the Senior Leadership in Animal Welfare and Management Committee of the Detroit Zoological Society.

### 2.1. Subjects and Housing

Three adult male gorillas were housed in a bachelor group at the Detroit Zoo, Royal Oak, MI, USA, in the Great Apes of Harambee habitat. The gorillas were captive-born, paternal half-siblings named Chip, Pende and Kongo and were 21, 20 and 19 years old, respectively, at the start of the study. There was a clear consensus from the animal care staff that Kongo was the most dominant individual, but there was some variation with the order between Chip and Pende. Gorillas were rotated between two large outdoor habitats or, when temperatures were below 1.7 °C, two indoor habitats. The gorillas were separated for approximately two hours each morning while their primary habitat was serviced and husbandry tasks were performed. Each gorilla had access to one or two rooms that measured 2.1 m wide × 3 m deep × 2.3 m high. The gorilla diet consisted of leafy greens, vegetables, fruit and low-starch biscuits, with additional browse or alfalfa hay provided on a daily basis. The morning diet was used to reinforce behaviors during data collection, and the remaining diet was fed out after data collection was completed.

### 2.2. Experimental Conditions

This study consisted of three conditions: control condition (no human interaction), cognitive task and training session. The order of the conditions was pseudo-randomized based on animal care staff scheduling needs. A single condition was tested each day, and each gorilla was tested on the same condition within a testing day. Testing for all three gorillas occurred from February to August 2018, starting between 7:00 a.m. and 8:00 a.m. and lasting approximately 90 min per testing day. Data collection took place while the gorillas were separated into individual rooms during routine morning husbandry. On occasion, a gorilla had access to two rooms if he did not readily shift. The testing order for the gorillas was randomized; however, there were occasional deviations from the order if a gorilla chose not to participate immediately. The gorillas were tested on each condition ten times, except for Chip, who chose not to participate for one training session and therefore completed nine training sessions. In addition to Chip not participating during one training session, he also chose not to provide saliva samples during eight testing days that spanned all three conditions. Behavioral data were also unavailable for one control session for Chip and Pende because they had access to two rooms, and video was not collected during one cognitive task session for Kongo. Exact sample sizes for each indicator are detailed in the results section.

**Figure 1:**
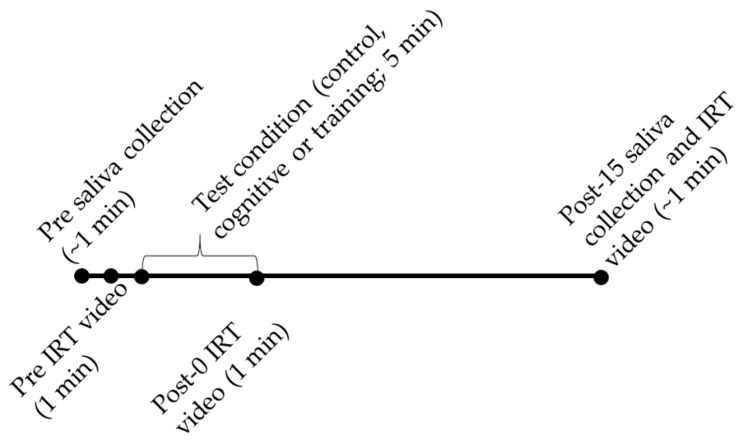
Timeline for data collection.

To explore the effects of HAIs, saliva samples and IRT video were collected before (pre) and after (post-0 or post-15) the three conditions to measure changes in indicators of welfare. Gorillas were not fed their morning diet prior to data collection to minimize the potential for food contamination during saliva collection. The gorillas were accustomed to receiving their morning meal about an hour late three days a week when they participated in ongoing cognition research, so this did not represent a major change in their routine. Data collection started with collecting pre-condition saliva samples and nasal temperatures (Figure 1). The primate supervisor collected saliva from the gorillas by having them thoroughly chew on two absorbent swabs (Salimetrics Oral Swab, Salimetrics LLC, State College, PA, USA) for about one minute total. This behavior had previously been trained using positive reinforcement, and the gorillas were very familiar with the procedure prior to the onset of data collection. Samples were immediately labeled and placed on ice until sample collection was completed for each day, at which point samples were processed in the lab at the Detroit Zoo. Next, the gorilla was stationed at the front of the mesh to record radiometric video to measure nasal temperatures for 1 min using an infrared thermography camera (T650sc, FLIR, Stockholm, Sweden; a temperature sensitivity of 0.02 K; a resolution of 640 × 480 pixels) with a 41.3 mm lens. The primate supervisor periodically reinforced the gorilla with small pieces of food to have the nose visible between the 5.08 cm × 5.08 cm gaps in the mesh. Each condition (described below) lasted for approximately 5 min and was videotaped (Vixia HFR400, Canon, Tokyo, Japan) for behavioral analysis. After each condition, nasal temperature was immediately measured for 1 min while the gorilla was stationed at the mesh (post-0). Saliva samples were collected 15 min after completion of the session (post-15). This collection time was selected because previous research demonstrated that oxytocin concentrations were elevated between 15–30 min following an intranasal oxytocin challenge [34], while changes in temperature measured via IRT occur almost instantaneously [36]. During the post-15 saliva collection, IRT video was also recorded while the gorillas were involved in saliva collection. The gorillas were at the mesh but were not stationed during the post-15 IRT video. Between the post-0 and post-15 measurements, the human participants did not directly interact with the focal gorilla or encourage him to engage in any specific task. However, the focal gorilla did have visual and auditory access to the humans, who conducted tests with other gorillas during this period.

In order to control for the effects of familiarity with the humans involved, the same individuals played consistent roles in each condition. A primate supervisor who has worked with these three gorillas for more than eleven years (including serving as the primary trainer for Kongo until around the midpoint of the study) served as the human interactor for all the training conditions. She also collected all the saliva samples and stationed the gorillas before and after all the conditions so that IRT video could be collected. Following several months of habituating the gorillas to his presence, the same researcher (M.H.) recorded all the IRT video. Finally, all cognitive tasks were administered by the same female external researcher who has worked with these gorillas for more than five years using a touchscreen interface.

#### 2.2.1. Control

The control condition consisted of only the interactions necessary to collect saliva and IRT samples. Immediately following the collection of pre-condition samples, the primate supervisor and researcher left the area, and the gorillas were left alone for five minutes, during which no human directly interacted with them. Normal animal care husbandry routines continued nearby, so the gorillas could hear and sometimes see animal care staff during this time period. Gorillas received food rewards during saliva collection and nasal temperature recording, so a small amount of food was occasionally available at the start of the five-minute period but was quickly consumed. The gorillas were not prompted to engage in any specific behaviors during this time, so the control lacked both the human and task-directed elements of the experimental conditions.

#### 2.2.2. Cognitive Task

The cognitive task for this study involved a simple memory task of touching a single clipart image on a monitor followed by receiving a food reward. After 27 trials, three test trials were conducted, which consisted of selecting three of the learned clipart images while ignoring six distractor images. Additional details on this testing paradigm are available in Vonk and Jett [45]. Gorillas were only rewarded if they selected the studied images. Cognitive tasks have been administered to the gorillas by this external researcher for over five years, and all the gorillas were already very familiar with this task at the onset of data collection. After completing each trial, there was an auditory tone, and the researcher dropped a piece of food down a chute. The researcher stood behind the computer cart, so she was only partially visible. However, she occasionally gave auditory cues to the gorillas to orient them to the cognitive task if needed. If this task was completed in under five minutes, it was repeated until the time expired. Pre, post-0 IRT samples and post-15 IRT and saliva samples were collected as previously described.

#### 2.2.3. Training Session

The training condition consisted of the primate supervisor performing a routine training session with an individual gorilla. Cues primarily consisted of the presentation of body parts and also included familiar medical devices (e.g., ultrasound probe). The training requests did not follow any order, rather were spontaneous and focused on keeping the focal gorilla engaged in the task. The primate supervisor used a clicker to audibly mark each time the gorilla performed the requested behavior, which served as a bridge for food rewards that were delivered on a variable reinforcement schedule. Again, pre, post-0 IRT samples and post-15 IRT and saliva samples were collected as described.

### 2.3. Infrared Thermography Analysis

**Figure 2:**
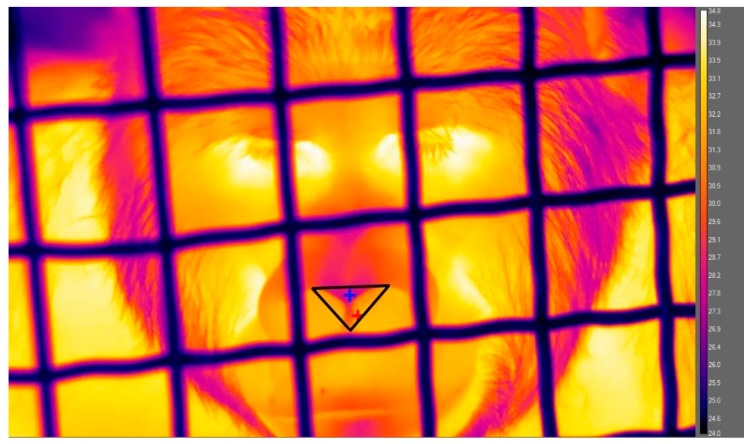
Example of a gorilla infrared thermography image. The black open triangle corresponds to the region of interest (ROI). The blue and red crosshairs inside the ROI correspond to the minimum and maximum temperatures, respectively. The color bar represents the surface temperature (°C).

The IRT videos were analyzed using FLIR ResearchIR Max (Version 4.40.6, FLIR, Stockholm, Sweden) by measuring temperatures within a region of interest (ROI; Figure 2) consisting of a triangle from the top of the nostrils to the bottom of the apex (the lowest point between the nostrils). ROIs were measured within optimal frames selected using the following criteria: (1) the image was not blurry, (2) the ROI was not blocked by any barriers, (3) the gorilla was not inhaling (causing a decrease in temperature inside the nostril) and (4) the face was between a frontal orientation and a 45° angle [42]. The minimum temperature was recorded for the ROI to avoid challenges with identifying a consistent physical landmark or having the ROI mean including a varying amount of pixels. Pre and post-0 videos were analyzed approximately every 30 s by selecting one optimal frame within each of the following three intervals: 0–10 s, 20–40 s, and 50–60 s. It was generally possible to identify a frame meeting these criteria because the gorillas were stationed in front of the mesh during IRT filming. We were only unable to identify a suitable frame in 4% of the total time intervals. In those cases, the remaining two time points were averaged, while all three time points were averaged when available to create a mean minimum temperature score for the minute. Temperature measurements were very consistent within each one-minute sample, with an average coefficient of variation between the three time points of 0.4%. For the post-15 IRT videos, only one optimal frame was selected because the gorillas were not stationed with their heads in a fixed location; however, the same frame selection criteria were still applied. The researcher who collected the videos coded all the IRT data but was blind to the condition being scored to avoid unintentional scoring biases.

### 2.4. Gorilla Behavior

BORIS software (v.7.4.7, Olivier Friard and Marco Gamba, Torino, Italy) [46] was used to code all occurrences of selected behavior events [47] during each condition. We used departures from the mesh to approximate the level of engagement the gorillas had in the HAIs [26]. The full ethogram is presented in Table 1. Reliability between two observers was assessed by calculating the percent difference in behaviors coded on a subset of videos that included all three conditions. Inter-observer reliability was greater than 90%.

### 2.5. Hormone Analysis

All saliva samples for the pre-condition measurements were collected between 7:02 a.m. and 8:48 a.m, and all the saliva samples for the post-15 measurements were collected between 7:26 a.m. and 9:05 a.m. However, paired samples were used for data analysis, and the interim between the pre and post-15 samples was 15–20 min within a pair. Following sample collection, saliva samples were centrifuged at 2500 rpm for 20 min at 4 °C to separate the saliva from the swab. Samples were then aliquoted into two parts to avoid repeated freeze-thaw cycles and stored at −20 °C. A sufficient amount of saliva was only collected consistently from Pende and Kongo to analyze both salivary oxytocin and cortisol, so only salivary cortisol was analyzed for Chip. All samples were processed and analyzed in the endocrinology laboratory at the Detroit Zoo.

#### 2.5.1. Salivary Cortisol

Unextracted saliva was used to measure cortisol using an enzyme immunoassay developed with commercially-sourced standard, enzyme conjugate and rabbit anti-cortisol primary antibody (ISWE002, Arbor Assays, Ann Arbor, MI, USA) and microtiter plates coated in house with goat anti-rabbit IgG secondary antibody. Saliva was diluted in assay buffer and analyzed in duplicate at a 1:8 dilution; however, some samples were run at additional dilutions (dilution range: 1:2 to 1:64) based on the percent binding (range: 20–80%). Serial dilutions (*n* = 8) of pooled gorilla samples showed parallel displacement to the standard curve (ANCOVA: F_1,13_ = 0.001, *p* = 0.97). Recovery values from pooled samples spiked with high (1600 pg/mL) and low standards (100 pg/mL) were 104% and 99%, respectively. A pooled gorilla sample was analyzed as a control on each cortisol plate. Inter- and intra-assay coefficients of variation were both below 15%. For both cortisol and oxytocin, endpoint values were measured using a BioTek Epoch spectrophotometer (BioTek Instruments, Winooski, VT, USA) at 450 nm, and concentrations were calculated using the plate reader’s four-parameter logistic curve software (Gen5^®^ v.2.06.10, BioTek Instruments, Winooski, VT, USA).

#### 2.5.2. Salivary Oxytocin

Unextracted saliva was used to measure oxytocin, as it has been shown to be an effective source [34]. Saliva was thawed, centrifuged again to remove any large particles or precipitates and evaporated under forced air using an evaporator manifold (Zipvap 20, #109A-11-80201, Glas-Col, Terre Haute, IN, USA) at 37 °C and reconstituted in oxytocin assay buffer. Oxytocin concentrations were measured using an enzyme immunoassay kit (DetectX^®^ K048, Arbor Assays, Ann Arbor, MI, USA). This kit was previously validated for gorilla salivary oxytocin in another study [34] and was validated in the Detroit Zoo lab by assessing parallelism and accuracy/recovery to test for matrix interference. Serial dilutions (*n* = 6) of pooled gorilla samples showed parallel displacement to the standard curve (ANCOVA: F_1,12_ = 3.07, *p* = 0.11). Recovery values from pooled samples spiked with a high standard (1600 pg/mL), medium standard (256 pg/mL) and low standard (40.96 pg/mL) were 113%, 112% and 99%, respectively. All samples were analyzed in duplicate at a neat dilution. A pooled gorilla sample was analyzed as a control on each oxytocin plate. Inter- and intra-assay coefficients of variation were both below 15%.

### 2.6. Statistical Methods

Statistical analyses were conducted in SPSS Version 25 (IBM Corporation, Armonk, NY, USA). Non-parametric Spearman correlations were used to examine correlations between sample collection time and hormone values. Rates of behaviors observed were calculated by dividing the count of each behavior by the length of each session. All statistical analyses were conducted within single individuals. Pre, post-0 and post-15 measurements of nasal temperature and salivary hormones were compared within conditions using Wilcoxon signed rank tests using testing days as the unit of analysis. To compare across conditions, changes in salivary cortisol and oxytocin were calculated by subtracting the pre concentration from the post-15 concentration, and changes in nasal temperature were calculated by subtracting the pre concentrations from the post-0 concentration. We did not compare the post-15 IRT measurements across conditions. Friedman tests were first used to examine if there was a difference within an individual among the three conditions, and follow-up pairwise comparisons were completed using Wilcoxon signed rank tests. All non-parametric tests were based on Monte Carlo sampling methods (10,000 permutations) with a 95% confidence interval due to the small sample size in this study [48]. This approach is similar to a randomization test, which is used to assess the probability that a certain set of responses occurred without making inferences about putative population values [48]. Therefore, the results of these analyses are meant to indicate whether responses to the conditions differed within each individual, but we do not make claims that these results apply to other gorillas. For this reason, we felt it was unnecessary to control for Type I error by applying a Bonferroni or similar correction because these corrections account for erroneous inferences about populations, which we did not attempt to make.

## 3. Results

### 3.1. Infrared Thermography

**Figure 3:**
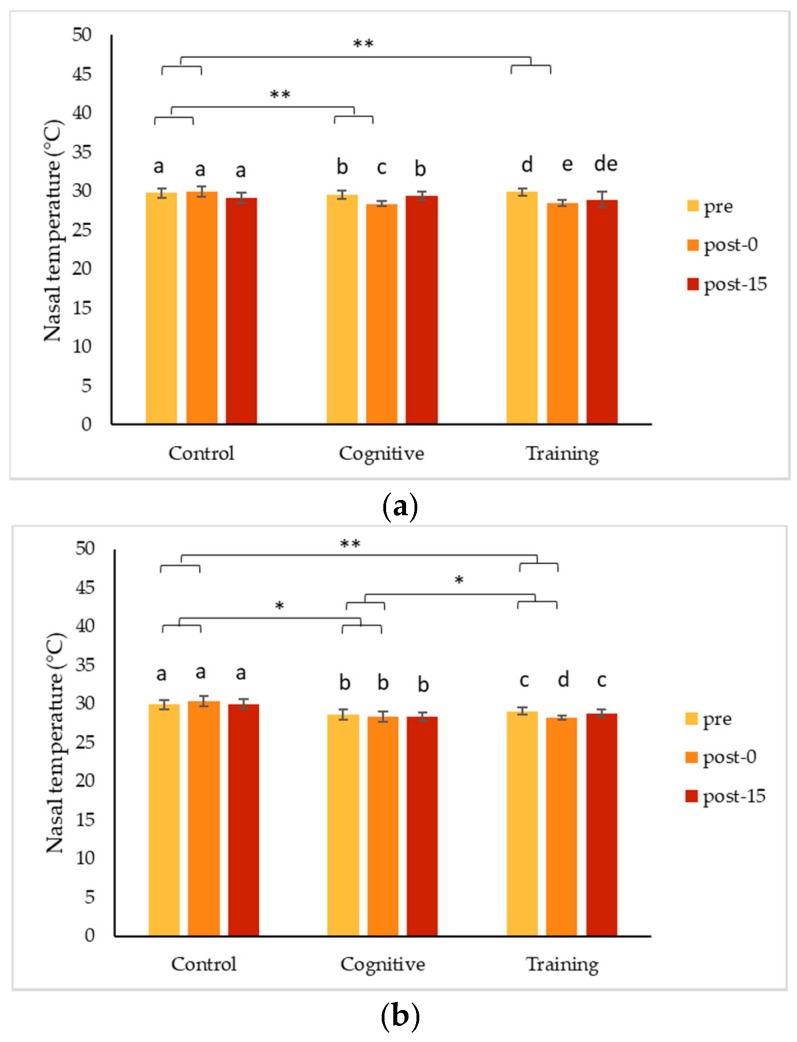

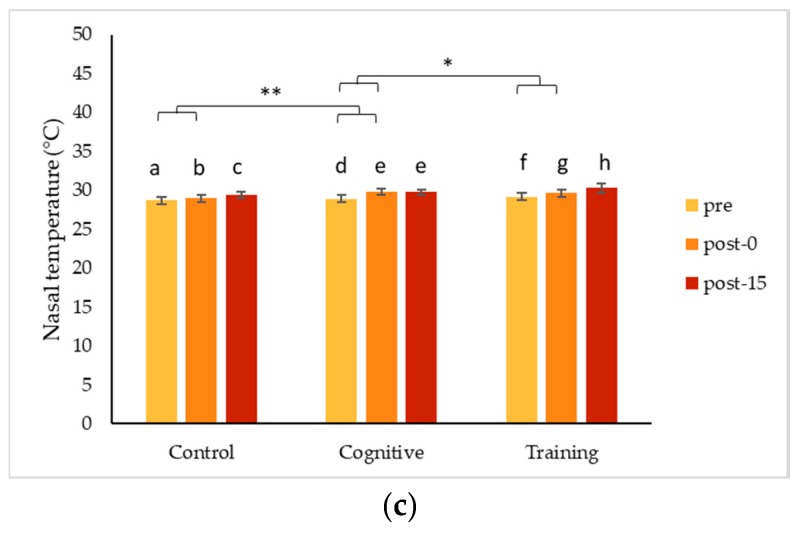
Minimum nasal temperatures (mean ± standard error) across three time points for two types of human–animal interactions and a control condition for (**a**) Chip; (**b**) Pende; and (**c**) Kongo. Brackets with ** indicate a significant difference of *p* < 0.05 and * indicate a trend (0.05 ≤ *p* < 0.1) for changes in temperature (calculated by subtracting pre from post-0 measurements) between conditions. Bars with different letters within a condition are significantly different or trending at *p* < 0.10 comparing time points within that condition. All tests were conducted using Wilcoxon signed rank tests with Monte Carlo sampling.

Paired pre to post-0 changes in minimum nasal temperature analyzed within a condition (control, training or cognitive) for each individual were variable (Table 2). The minimum nasal temperature decreased significantly from baseline for Chip following both the HAI conditions (training and cognition) but returned to pre-condition values after 15 min in each case (Figure 3a, Table 2). Chip’s minimum nasal temperature increased slightly following the control condition, but this change was not statistically significant. Pende demonstrated a decrease in nasal temperature, which returned to pre-condition levels after 15 min, following the training session but did not show a significant temperature change in either the cognitive or the control condition (Figure 3b). Conversely, the minimum nasal temperature for Kongo increased following the control condition, cognitive task and training session; and it remained elevated after 15 min in each condition as well (Figure 3c).

Comparing changes in temperature (based on the difference from pre to post-0 measurements) among the three conditions (Figure 3), nasal temperatures decreased following the human interactions (training sessions and cognitive task) but increased after the control condition for two gorillas, while the third gorilla showed an increase in nasal temperature following all three conditions. Nasal temperature decreased more for Chip following the cognitive task (Wilcoxon signed rank test, *n* = 10, z = −2.395, *p* = 0.014) and training session (Wilcoxon signed rank test, *n* = 9, z = −0.666, *p* = 0.004) compared to the control condition. However, there was no difference in the magnitude of nasal temperature changes between the cognitive task and training session (Wilcoxon signed rank test, *n* = 9, z = −0.889, *p* = 0.434) for Chip. Pende’s nasal temperature decreased significantly during the training session compared to the control condition on average (Figure 3; Wilcoxon signed rank test, *n* = 10, z = −2.497, *p* = 0.009). Pende also showed trends for his nasal temperature to decrease by a greater magnitude following the training session compared to the cognitive task (Wilcoxon signed rank test, *n* = 10, z = −1.683, *p* = 0.099) and to decrease more following the cognitive task compared to the control condition (Wilcoxon signed rank test, *n* = 10, z = −1.886, *p* = 0.065). Lastly, Kongo experienced a significantly greater increase in minimum nasal temperature during the cognitive task compared to the control condition (Figure 3; Wilcoxon signed rank test, *n* = 10, z = −2.710, *p* = 0.005) and a trend for greater increase during the cognitive task compared to the training session (Wilcoxon signed rank test, *n* = 10, z = −1.886, *p* = 0.059). However, the magnitude of increase in nasal temperature did not differ statistically for Kongo comparing the training session and control condition (Wilcoxon signed rank test, *n* = 10, z = −0.663, *p* = 0.539).

### 3.2. Gorilla Behaviors

Chip, Pende and Kongo consumed food at a mean rate of 1.826 (±0.501), 1.770 (±0.586) and 2.137 (±0.160) items/min, respectively, during training sessions. They consumed food during the cognitive task at a mean rate of 4.709 (±1.291), 6.141 (±0.948) and 8.568 (±0.950) items/min for Chip, Pende and Kongo, respectively.

**Figure 4:**
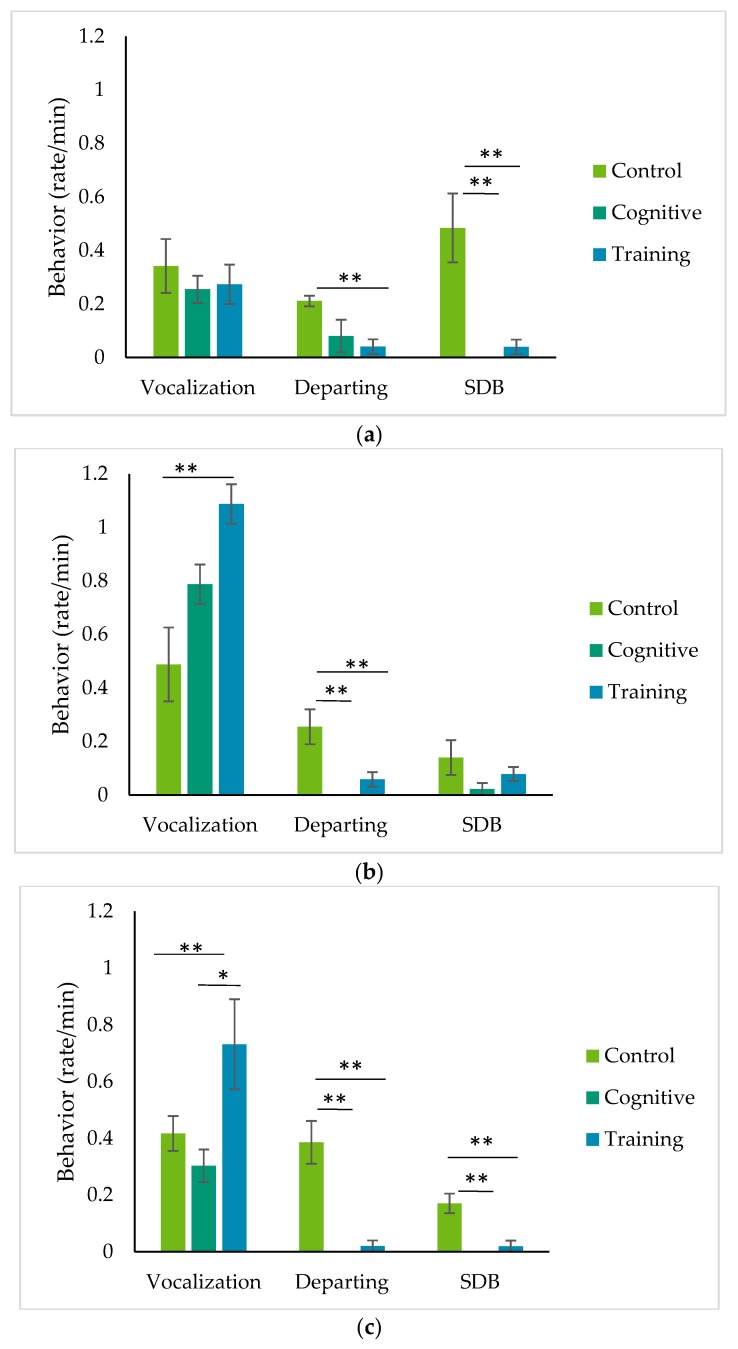
Rates of all-occurrence behaviors (mean ± standard error) including grumble vocalizations (vocalization), mesh depart (departing) and self-directed behavior (SBD) during two types of human–animal interactions and a control condition for (**a**) Chip; (**b**) Pende; and (**c**) Kongo. ** Indicates a significant difference of *p* < 0.05 and * indicates a trend (0.05 ≤ *p* < 0.1) between conditions connected by a line based on Wilcoxon signed rank tests with Monte Carlo sampling.

The rate of departing from the mesh was higher during the control condition compared to the training session for Chip (Figure 4; Wilcoxon signed rank test, *n* = 8, z = −2.521, *p* = 0.009), but there were no differences in rates of departing between the control condition and the cognitive task (Wilcoxon signed rank test, *n* = 9, z = −1.599, *p* = 0.128) or the cognitive task and the training session (Wilcoxon signed rank test, *n* = 9, z = −1.342, *p* = 0.491). Both Pende and Kongo had higher departing rates during the control condition compared to the cognitive task (Wilcoxon signed rank test, Pende: *n* = 9, z = −2.666, *p* = 0.003; Kongo: *n* = 9, z = −2.521, *p* = 0.009) and the training session (Wilcoxon signed rank test, Pende: *n* = 10, z = −2.547, *p* = 0.006; Kongo: *n* = 9, z = −2.547, *p* = 0.009). However, there was no difference in departing for Pende and Kongo between the cognitive task and the training session (Wilcoxon signed rank test, Pende: *n* = 10, z = −1.342, *p* = 0.500; Kongo: *n* = 9, z = −1.000, *p* = 1.000). Neither Pende nor Kongo departed from the mesh during the cognitive sessions.

The rate of SDB did not differ for Pende among any of the conditions (Figure 4; Friedman test, *n* = 9, df = 2, χ^2^ = 2.100, *p* = 0.423), so no pairwise comparisons were made between conditions. SDB was higher for Chip and Kongo in the control condition compared to both the cognitive task (Wilcoxon signed rank test, Chip: *n* = 9, z = −2.521, *p* = 0.007; Kongo: *n* = 9, z = −2.366, *p* = 0.015) and the training session (Wilcoxon signed rank test, Chip: *n* = 8, z = −2.336, *p* = 0.016; Kongo: *n* = 10, z = −2.380, *p* = 0.017). However, there was no difference in SDB for Chip and Kongo between the cognitive task and the training session (Wilcoxon signed rank test, Chip: *n* = 9, z = −1.342, *p* = 0.502; Kongo: *n* = 9, z = −1.000, *p* = 1.000).

The rate of grumble vocalizations did not differ for Chip for any of the conditions (Figure 4; Friedman test, *n* = 8, df = 2, χ^2^ = 0.194, *p* = 0.938), so no pairwise comparisons were made between conditions. Grumble vocalizations were higher for Pende and Kongo in the training session compared to the control condition (Wilcoxon signed rank test, Pende: *n* = 9, z = −2.547, *p* = 0.006; Kongo: *n* = 10, z = −2.090, *p* = 0.040) and there was a trend for grumble vocalizations to be higher in the training session compared to the cognitive task for Kongo (Wilcoxon signed rank test, *n* = 9, z = −1.960, *p* = 0.058). However, there was no difference in grumble vocalizations for Pende and Kongo between the control condition and the cognitive task (Wilcoxon signed rank test, Pende: *n* = 9, z = −1.599, *p* = 0.130; Kongo: *n* = 9, z = −0.770, *p* = 0.497) or between the cognitive task and the training session for Pende (Wilcoxon signed rank test, *n* = 10, z = −1.682, *p* = 0.106).

### 3.3. Salivary Cortisol

**Figure 5:**
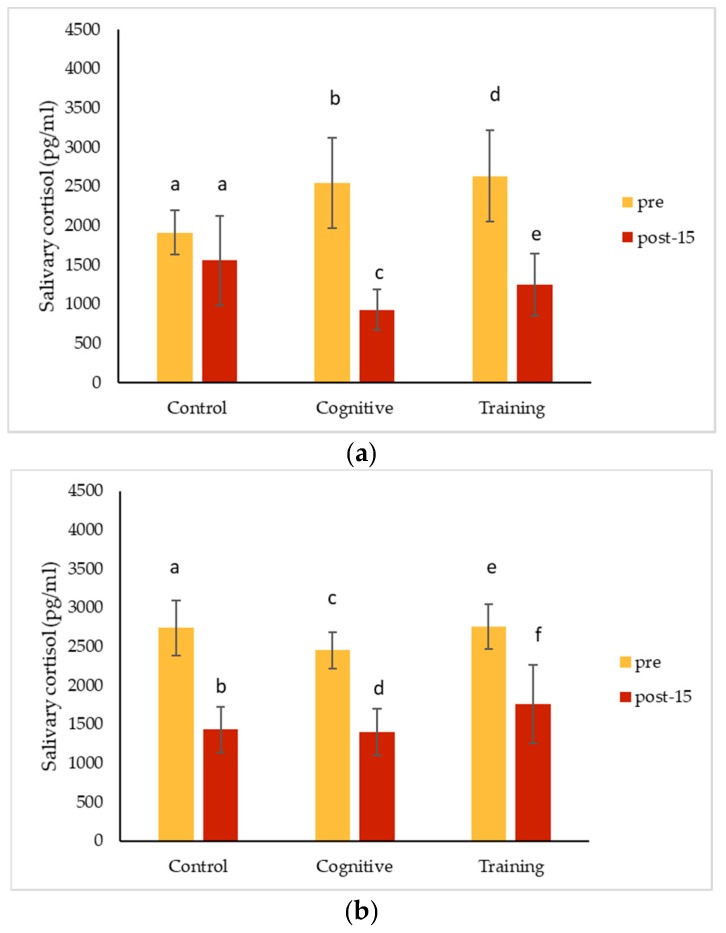

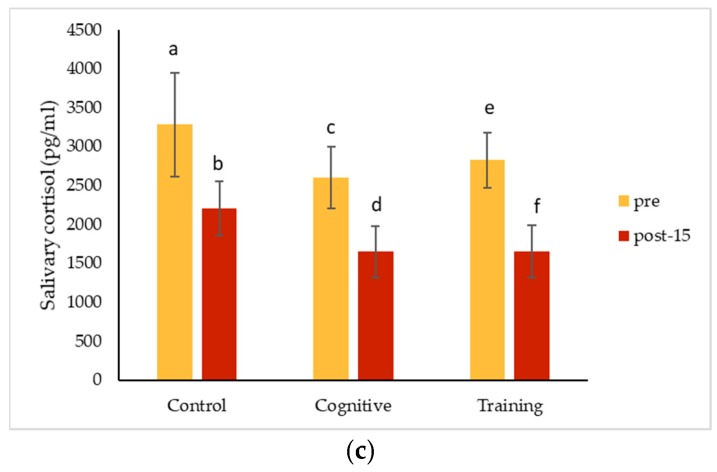
Salivary cortisol (mean ± standard error) before and after two types of human–animal interactions and a control condition for (**a**) Chip; (**b**) Pende; and (**c**) Kongo. Friedman tests comparing the change in hormone levels (calculated by subtracting the pre from the post-15 values) across conditions were non-significant for each gorilla. Bars with different letters within a condition are significantly different or trending at *p* < 0.10 comparing time points within that condition based on Wilcoxon signed rank tests with Monte Carlo sampling.

Salivary cortisol decreased significantly from pre-condition concentrations for all three gorillas following the control condition, cognitive task and training session (Table 2; Figure 5). The change in salivary cortisol was not significantly different among conditions for Chip at the post-15 measurement (Friedman test, *n* = 5, df = 2, χ^2^ = 0.400, *p* = 0.953). Pende also did not have a significant difference in the magnitude of cortisol change across conditions (Friedman test, *n* = 10, df = 2, χ^2^ = 0.800, *p* = 0.709). Likewise, there was no significant difference in cortisol change comparing conditions for Kongo (Friedman test, *n* = 10, df = 2, χ^2^ = 0.200, *p* = 0.975). Overall (including samples from all three gorillas), salivary cortisol concentrations decreased with sample collection time (*n* = 160, Spearman’s ρ = −0.289, *p* < 0.001).

### 3.4. Salivary Oxytocin

**Figure 6:**
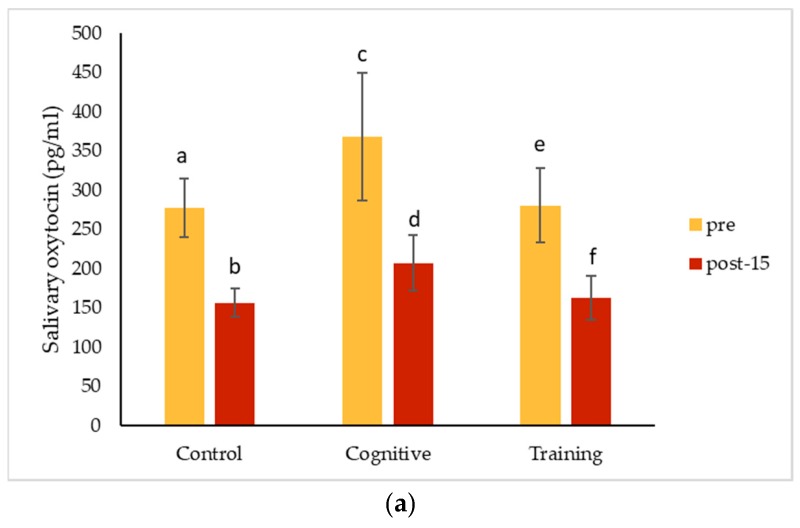

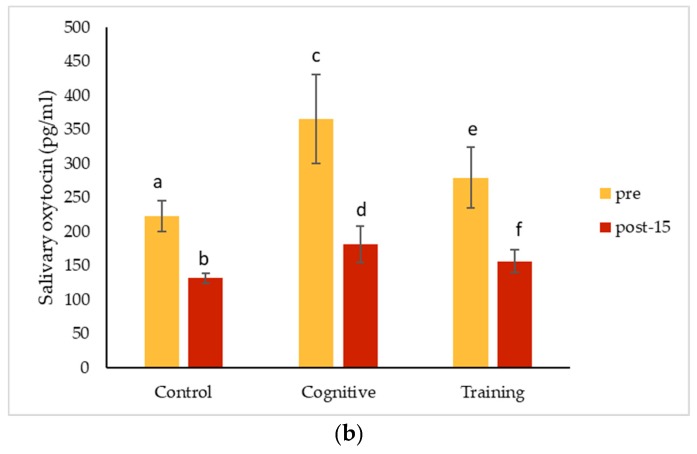
Salivary oxytocin (mean ± standard error) before and after two types of human–animal interactions and a control condition for (**a**) Pende and (**b**) Kongo. Friedman tests comparing the change in hormone levels (calculated by subtracting the pre from the post-15 values) across conditions were non-significant for each gorilla. Bars with different letters within a condition are significantly different or trending at *p* < 0.10 comparing time points within that condition based on Wilcoxon signed rank tests with Monte Carlo sampling.

Salivary oxytocin consistently decreased significantly from pre-condition values for both Pende and Kongo following the control condition, cognitive task and training session (Table 2; Figure 6). There was no significant difference in the magnitude of oxytocin decline among the three conditions for Pende (Friedman test, *n* = 10, df = 2, χ^2^ = 0.200, *p* = 0.971) or Kongo (Friedman test, *n* = 10, df = 2, χ^2^ = 0.200, *p* = 0.972). Oxytocin concentrations did not change significantly with sample collection time (*n* = 120, ρ = −0.145, *p* = 0.115).

## 4. Discussion

We tested the effectiveness of IRT for measuring the emotional responses of three gorillas to two types of interactions with familiar humans that are fairly common in zoos: positive reinforcement training and cognitive research tasks. We also collected observations of behavior and salivary endocrine samples for the purpose of interpreting the changes in arousal detected by IRT. As hypothesized, changes in nasal temperatures following HAIs indicated a change in arousal for all three gorillas, although temperatures decreased for two gorillas and increased for the third. Consistent with our predictions, salivary cortisol concentrations decreased for all the gorillas following HAIs; however, they also decreased following a control condition. Contrary to our prediction, salivary oxytocin decreased after all three conditions. The consistency among conditions suggests that the interactions did not exert a measurable effect on salivary hormone levels, or that methodological issues precluded detecting such an effect. Behaviorally, the gorillas showed a high level of engagement with the tasks, vocalized positively and showed few potentially negative indicators of welfare, as we predicted.

Although the nature of this investigation precludes extrapolating these results to other gorillas, it is notable that the responses detected by IRT were highly consistent within each individual, suggesting that the IRT method did show good reliability. Additionally, IRT responses differed between the control and interaction conditions, suggesting good construct validity—a conclusion that is tempered by the opposing direction of these changes within the sample of three gorillas. Additionally, the individual differences in IRT data did not co-vary reliably with the endocrine or behavioral measures; however, challenges associated with these other methods (including their construct validity) preclude any firm conclusions on this point. In general, poor correspondence among measures is one of the many challenges associated with measuring welfare. This exploratory investigation suggests that IRT has potential for understanding the response of nonhuman animals to interactions in a zoo setting, but further validation is needed before IRT can be applied in the absence of other contextual measures.

### 4.1. Changes in Nasal Temperature Following HAIs

Two of the gorillas, Chip and Pende, experienced a decrease in nasal temperature during the HAI conditions that contrasted significantly with the increase in nasal temperature they showed following the control condition. Unlike both his brothers, Kongo’s nasal temperature increased during all conditions, especially the cognitive task. Although these changes indicate a change in arousal, the differences in the directionality of the changes make it less clear as to what type of emotional response was being expressed. To date, IRT studies have largely been based on the assumption that a particular type of event is either positive (e.g., play) or negative (e.g., teasing), and that the arousal measured via IRT indicates an emotional response consistent with the presumed valence of the event. Much of the published literature on non-human primates reports decreases in nasal temperatures in response to presumed negative stimuli (e.g., [41,42,49]). However, decreases have also been seen in response to putatively positive stimuli in other species, including cows (*Bos Taurus*) [50] and humans [51,52]. Additionally, an increase in nasal temperature has been found following a positive experience in a rhesus macaque [43] and in humans when viewing images rated as positive [53]. Although more research has focused on measuring temperature decreases during increased arousal, blood flow changes corresponding to increased forehead temperatures have been observed in humans during relaxing activities such as meditation [54]. Measuring temperature in multiple locations, as suggested by Chotard et al. [55], and examining the changes observed in conjunction with one another may help to tease apart some of these conflicting results. It is also possible that in the case of these three gorillas, the interactions themselves did not result in emotional changes and that the differences seen in nasal temperatures are reflective of other factors.

Changes detected using IRT tend to be transitory in nature [42], and nasal temperatures returned to pre-condition levels within fifteen minutes for both Chip and Pende. Interestingly, this was not the case for Kongo, whose nasal temperature increased following HAIs and remained significantly elevated fifteen minutes later. The lack of consistency in the responses among the three gorillas presents a challenge when assessing the usefulness of IRT in the present study. However, individual differences in arousal measured via IRT have been found [56], and, in the current study, temperature responses were highly consistent within an individual, suggesting they reflect meaningful individual differences in an underlying physiological process. Based on our results, changes in temperature measured via IRT indicated changes in arousal, and information on the context or additional measures are needed to understand the valence of responses.

### 4.2. Patterns of Behavior during HAIs

Observations of the gorillas’ behavior during HAIs suggest that the gorillas were engaged with the tasks that structured these interactions. Once they began the HAI tasks, the gorillas seldom broke away, as evidenced by the lower rates of departing from the mesh during HAIs compared to the control condition for Kongo and Pende. For Chip, the difference between rates of departing the mesh was statistically significant comparing the control condition and the training task, but not comparing the control and cognitive conditions. However, the IRT data suggest that Chip’s level of arousal increased for both HAI conditions and not the control. Departure from the training area has been used a metric of participation in hamadryas baboons [26], but perhaps future studies could try other metrics. A recent review by Patel et al. [57] suggested measuring latency to shift into holding rooms for HAIs, or measuring the response accuracy (cognitive task) or latency to respond to cues (cognitive or training tasks) to capture the engagement of participants. In this study, the relative simplicity of the cognitive task (simply touching a single image on a screen) precluded further analyses examining how response accuracy affected the gorillas’ levels of engagement. Additionally, measuring the behavior of the humans involved in the interactions (including rates of verbal cues and reinforcement) may help explain variability in animal engagement, in addition to facilitating more systematic comparisons of how animals respond to different types of interactions.

Self-directed behavior is well-established as an indicator of anxiety in nonhuman primates (e.g., [15]) and has been linked to performance in chimpanzees and gorillas (incorrect versus correct trials; [58]), as well as task difficulty in chimpanzees [59]. Given that Chip broke away from the cognitive task more than the other gorillas, while Kongo completed the most cognitive trials, we might have expected Chip to show the highest rates of SDB during cognitive trials and Kongo to show the lowest. However, both individuals performed significantly more SDB during the control condition. These results could suggest that the relatively simple training and cognitive tasks we used did not provoke much anxiety, or could reflect individual differences in the response to testing anxiety, as reported in chimpanzees [59]. Alternatively, unstructured time in the holding rooms during the control condition could have led to greater anxiety or an anticipatory response related to returning to the habitat or other aspects of the morning routine. Finally, more SDB could have occurred during the control condition because opportunities to perform other behaviors were somewhat limited in the holding rooms; in another study, gorillas performed more SDB in holding compared to on exhibit, although the difference did not quite reach statistical significance [60].

Finally, two of the gorillas exhibited different rates of grumble vocalizations among the conditions. Grumbles are close-range calls used in multiple contexts, including foraging, travel and rest [61] and while the caller is performing relaxed behaviors [61]. In this case, grumbles could not have been related solely to food consumption because the gorillas vocalized more during the PRT sessions but ate more during the cognitive sessions. Salmi and colleagues [62] have suggested that these more generalized vocalizations may differ in the type of information they convey, such as arousal, depending on the context in which they are being used. Only two other vocalizations were identified during any of the conditions. These were a hoot and a grunt, which are not often associated with negative contexts [57].

In general, the limited variety of behaviors expressed by the gorillas during the study conditions created a methodological challenge. Our ethogram originally included undesirable behaviors, such as hair-plucking and regurgitation and reingestion, but we never observed these behaviors. We also coded rates of spontaneous object play and displays, but these occurred rarely and could not be tested statistically. The lack of negative indicators of welfare, coupled with the performance of positive vocalizations and the low rates of departing the mesh, suggest the HAIs may have been positive experiences for these gorillas. However, more behavioral indicators, particularly of positive emotions, would strengthen this interpretation. Studies of cognitive testing have also tended to focus on SDB or other potentially negative indicators, while assumptions about the positive effects of participation in cognitive testing on the animals’ sense of agency or experience of flow remain largely untested [9]. Future studies could deal with these challenges by employing some of the previously mentioned measures of latency [57] or by collecting behavioral data immediately before or after HAIs. Collecting data after the gorillas were reunited in their habitat could have detected any carryover effects of the HAIs on their social interactions, similar to the increases in affiliative and decreases in agonistic behaviors observed in ring-tailed lemurs (*Lemur catta*) following separation for positive reinforcement training sessions [63].

### 4.3. Hormonal Changes Accompanying HAIs

Salivary cortisol concentrations decreased significantly from pre-condition measurements for all conditions for Kongo and Pende, and after all conditions except for the control for Chip. Salivary oxytocin decreased for all individuals for each condition. The magnitude of these changes did not differ among the three study conditions for any individual. The almost universal decreases in salivary hormones across conditions could suggest that all the conditions affected the gorillas the same way. It is possible that the interactions necessary to collect pre-condition IRT and saliva samples caused an effect that carried over into the control condition, or that the effects of separating the gorillas for sample collection impacted measurements. Pair-bonded titi monkeys (*Callicebus cupreus*) showed an increase in cerebrospinal fluid oxytocin following short-term separation [64], and urinary oxytocin levels increased in cotton-top tamarin (*Saguinus oedipus*) pairs when they were reunited following separation [65]. Male gorillas in bachelor groups, such as the individuals in this study, form strong social bonds and actually have higher urinary oxytocin levels compared to males living in mixed-sex groups or alone [35]. Beginning data collection as soon as animals are separated or testing animals in a social setting (which presents its own challenges) may help avoid this potential pitfall in future studies. These hormone changes could also reflect an anticipatory response related to testing, which could have been cued by the delay in food presentation on cognitive testing days. Future assessments could begin data collection immediately after the gorillas are separated to avoid potential anticipatory responses. Additional methodological factors must also be considered as alternative explanations for our results.

Overall (grouping all conditions and subjects), salivary cortisol concentrations decreased significantly in samples collected later during the two-hour period the gorillas spent in the holding rooms. This finding is consistent with other studies in great apes. Kuhar et al. [66] started sample collection at 9:00 a.m. and found a steady decrease in salivary cortisol in gorillas until about 4:00 p.m. Although we are not aware of published data for the early morning from gorillas, salivary cortisol in chimpanzees declines steadily after 6:00 a.m. [67], suggesting levels in our study subjects may have been on the decline during the period we collected data. We timed our study to collect data during the morning when the gorillas are normally in their holding rooms for husbandry and cleaning purposes, but future studies may want to consider collecting data during the late afternoon when cortisol levels are more stable [66]. We did not find a statistical correlation between sample collection time and oxytocin values, which is consistent with the findings from Leeds [35]. However, Leeds [35] did find significantly lower urinary oxytocin levels in the afternoon compared to the morning, so timing should be taken into account when measuring oxytocin as well.

The effect of food contamination on salivary hormone measurements could also explain the decreases we saw across conditions. As noted, the gorillas received food rewards in all of the study conditions, not just the HAIs, because it was necessary to reward them for providing saliva samples and stationing at the mesh to collect IRT video. Flavoring of collection media is often necessary to gain compliance for sample collection from nonhuman primates and may (e.g., [68]) or may not (e.g., [27]) affect measured hormone concentrations. More controlled studies are possible with humans but still have shown mixed results, possibly due to the type of food contaminant and hormone assay used [69,70] or measuring other steroids [71]. There is less information for oxytocin or other peptides, but one study found that salivary oxytocin concentrations increased significantly when measured immediately after domestic dogs consumed treats [29]. Because it is difficult to avoid food during typical zoo HAIs, we suggest that in future studies, researchers control for possible contamination by consistently using a single type of food as a reward and providing access to food in equal intervals prior to collection of all samples [69]. Validation studies with human volunteers can also be used to assess the level of interference caused by the specific food utilized (e.g., [72]).

Although we encountered methodological difficulties in our aim to use salivary cortisol as a measure of the response to HAIs, we are not alone in finding mixed results. Participation in PRT is associated with a decrease in baseline salivary cortisol in hamadryas baboons, but pre-post comparisons within training sessions did not show a significant change [26]. Leeds [35] found a reduction in salivary cortisol following PRT in a single gorilla, while salivary cortisol did not change during PRT for orangutans and bonobos [27]. Similarly, Fagot et al. [73] reported lower salivary cortisol levels for Guinea baboons (*Papio papio*) during periods when they had access to cognitive testing, while another study found a decrease in salivary cortisol during testing for a single orangutan, despite concurrent increases in SDB and other behavioral signs of frustration [74]. If an animal is not already experiencing heightened adrenal activity in response to stress, there may be no need for cortisol to decrease during HAIs. This type of “floor effect” has also been identified as a challenge to studies of enrichment using cortisol as a dependent measure [75]. Another potential explanation for these mixed effects is that changes in cortisol or other glucocorticoids (GCs) may reflect arousal in response to either positive and negative events [76]. In this sense, GCs and IRT share a common ambiguity, in that each measure reflects arousal without providing information on whether that arousal represents a positive or negative emotional response.

Cortisol also may not be the ideal choice for interpreting the valence of temperatures detected via IRT because changes in nasal temperature reflect activation of the SNS, but changes in cortisol correspond to changes in the HPA axis. Kano and colleagues [42] found that two SNS measures, nasal temperature and heart rate variability, in chimpanzees decreased after conspecific playbacks, but cortisol did not change. Subsequent HAI studies could examine how changes in surface temperature correspond to other SNS biomarkers, including heart rate and alpha-amylase [77]. However, there are some methodological challenges associated with measuring salivary alpha-amylase (sAA) due to its role in the enzymatic digestion of starch, which means its release is strongly affected by salivary flow rate, chewing behavior and food ingestion [78].

In the quest for positive indicators of welfare, oxytocin has recently received a lot of attention. In this study, oxytocin concentrations measured in saliva for Pende and Kongo declined following both the HAI and control conditions. Together with Leeds [35], changes in oxytocin related to HAIs have only been studied in four gorillas, and neither study found an effect of PRT on this measure. Clearly additional data are needed, but it is also possible that interacting with humans does not affect oxytocin levels in western lowland gorillas. Instead, we may need to critically examine the assumption that increased oxytocin reflects positive emotions [33], especially in contexts that are not ecologically-relevant to animals. After all, numerous studies have supported the hypothesis that oxytocin plays a role in regulating non-reproductive affiliative behavior between conspecifics [79], but it is possible that this system does not regulate interactions occurring across species.

## 5. Conclusions

For this study, we attempted to validate the use of IRT to measure the emotional responses of gorillas to interactions with familiar humans by comparing IRT results with behavioral and endocrine measures. However, the lack of consistency among these measures, as well as the clear difference between subjects in the directionality of nasal temperature changes, made interpreting these findings difficult. Due to the small sample size and the variability in responses, we also cannot rule out the possibility that the HAIs did not have a meaningful effect on the gorillas at all, or that none of the measures we used could detect those effects. We have identified methodological issues that may have affected the validity of hormone measurements (e.g., food contamination and circadian variation), as well as challenges in finding useful behavioral measures during structured interactions. However, it is likely that at least some of the discrepancies among our measures were due to other factors inherent to the nature of HAIs.

The multifaceted nature of HAIs is a major challenge for studying their effects on animals. In this study, HAIs involved interacting with humans, performing cognitive tasks and learned behaviors, as well as receiving food rewards from humans. Minimal food was available to the gorillas in the control condition, so, in this study, the control really represented the near absence of all three of these factors. Any of these factors could be related to the changes in arousal that were detected, and it is not necessarily the case that each gorilla responded to the same component of the HAI. The difference in the availability of food rewards between the three conditions also made it impossible to differentiate whether the responses we observed were due to food availability or other aspects of the interactions. For now, researchers may want to simplify the types of interactions used in these studies to isolate these factors. Although food consumption is a major confound, most HAIs between animals and animal care staff in zoos involve food. To address the issue of food consumption, a control condition could consist of simply feeding the animals, rather than avoiding interactions altogether. Confounds related to social separation, amount of space available during testing (in this study, one vs. two holding rooms), human familiarity and individual personality also require careful consideration.

Overall, our behavioral data suggest that the interactions with humans were likely positive experiences for the gorillas in this study, although additional behavioral measures would have strengthened this interpretation. The benefits of HAIs such as PRT could be related to building social bonds with humans, experiencing a sense of agency [80] and/or control [8] or simply providing variety and change in the routine [81]. Clarifying the nature of the emotional response to interactions could help differentiate between these potential benefits and is also important for understanding how these interactions affect the welfare of the animals involved. Although we encountered many challenges in our attempt to measure emotional responses in gorillas, continued research that encompasses an emotional component is necessary for gaining a comprehensive view of animal welfare.

## Figures and Tables

**Table 1:** Gorilla ethogram for all-occurrence behaviors.

Behavior	Operational Definition
Eat	Focal consumes edible items
Mesh depart	Focal moves at least one body width away from the mesh
Self-directed behavior	Focal uses the fingers or lips to comb or pick through hair or makes repeated raking motions with the fingers against the body
Grumble vocalization	Focal produces soft, rumble-like noise

**Table 2:** Pre-post comparisons following human–animal interactions (cognitive task or training session) and a control condition for three western lowland gorillas.

Condition: Comparison	Chip			Pende			Kongo		
	*n*	*Z*	*p*-Value	*n*	*Z*	*p*-Value	*n*	*Z*	*p*-Value
Control									
IRT pre–post-0	10	−2.497	0.573	10	−1.021	0.236	10	−2.701	0.027 ** ↑
IRT pre–post-15	9	−1.260	0.823	10	−0.770	0.861	10	−2.395	0.004 ** ↑
IRT post-0–post-15	9	−1.680	0.110	10	−0.764	0.492	10	−2.397	0.012 ** ↑
Oxytocin pre–post-15	-	-	-	10	−2.293	0.019 ** ↓	10	−2.701	0.004 ** ↓
Cortisol pre–post-15	7	−1.014	0.382	10	−2.803	0.002 ** ↓	10	−2.090	0.037 ** ↓
Cognitive task									
IRT pre–post-0	10	−0.612	0.010 ** ↓	10	−1.274	0.332	10	−2.142	0.004 ** ↑
IRT pre–post-15	8	−0.296	0.244	10	−0.204	0.495	10	−2.701	0.014 ** ↑
IRT post-0–post-15	8	−2.521	0.008 ** ↑	10	−0.510	0.647	10	−0.102	0.926
Oxytocin pre–post-15	-	-	-	10	−1.988	0.049 ** ↓	10	−2.191	0.028 ** ↓
Cortisol pre–post-15	8	−2.521	0.007 ** ↓	10	−2.497	0.010 ** ↓	10	−2.803	0.002 ** ↓
Training session									
IRT pre–post-0	9	1.000	0.004 ** ↓	10	−2.805 **	0.002 ** ↓	10	−2.395	0.014 ** ↑
IRT pre–post-15	6	−0.314	0.842	10	−1.020	0.337	9	−2.556	0.008 ** ↑
IRT post-0–post-15	6	−1.153	0.311	10	−1.887	0.059 * ↑	9	−2.312	0.021 ** ↑
Oxytocin pre–post-15	-	-	-	10	−1.886	0.064 * ↓	10	−2.497	0.010 ** ↓
Cortisol pre–post-15	5	−2.310	0.061 * ↓	10	−1.988	0.049 ** ↓	10	−2.803	0.002 ** ↓

Note. Infrared thermography nasal temperatures, salivary oxytocin and salivary cortisol were compared before and after (either immediately after for “post-0” or fifteen minutes later for “post-15”) the conditions using a Wilcoxon signed rank test with Monte Carlo sampling. Asterisks indicate level of significance with ** representing *p*-values < 0.05 and * showing a trend for 0.05 ≤ *p* < 0.1. Direction of the change is denoted with an arrow. All three gorillas were tested in all conditions 9–10 times. However, not all measures could be collected at each trial, so exact *n* values for each test are listed. IRT: infrared thermography.

## References

[1] Ward S.J., Sherwen S., Clark F.E. (2018). Advances in applied zoo animal welfare science. J. Appl. Anim. Welf. Sci..

[2] Hosey G., Melfi V. (2014). Human-animal interactions, relationships and bonds: A review and analysis of the literature. Int. J. Comp. Psychol..

[3] Hemsworth P.H. (2003). Human–animal interactions in livestock production. Appl. Anim. Behav. Sci..

[4] Barker S.B., Wolen A.R. (2008). The benefits of human–companion animal interaction: A review. J. Vet. Med. Educ..

[5] Hosey G., Birke L., Shaw W.S., Melfi V. (2018). Measuring the strength of human–animal bonds in zoos. Anthrozoös.

[6] Smith J.J. (2014). Human–animal relationships in zoo-housed orangutans (*P. abelii*) and gorillas (*G. g. gorilla*): The effects of familiarity. Am. J. Primatol..

[7] Egelkamp C.L., Ross S.R. (2019). A review of zoo-based cognitive research using touchscreen interfaces. Zoo Biol..

[8] Claxton A.M. (2011). The potential of the human–animal relationship as an environmental enrichment for the welfare of zoo-housed animals. Appl. Anim. Behav. Sci..

[9] Clark F.E. (2017). Cognitive enrichment and welfare: Current approaches and future directions. Anim. Behav. Cognit..

[10] Paul E.S., Mendl M.T. (2018). Animal emotion: Descriptive and prescriptive definitions and their implications for a comparative perspective. Appl. Anim. Behav. Sci..

[11] Anderson D.J., Adolphs R. (2014). A framework for studying emotions across species. Cell.

[12] Boissy A., Manteuffel G., Jensen M.B., Moe R.O., Spruijt B., Keeling L.J., Winckler C., Forkman B., Dimitrov I., Langbein J. (2007). Assessment of positive emotions in animals to improve their welfare. Physiol. Behav..

[13] Leeds A., Elsner R., Lukas K.E. (2016). The effect of positive reinforcement training on an adult female western lowland gorilla’s (*Gorilla gorilla gorilla*) rate of abnormal and aggressive behavior. Anim. Behav. Cognit..

[14] Carrasco L., Colell M., Calvo M., Abelló M.T., Velasco M., Posada S. (2009). Benefits of training/playing therapy in a group of captive lowland gorillas (*Gorilla gorilla gorilla*). Anim. Welf..

[15] Maestripieri D., Schino G., Aureli F., Troisi A. (1992). A modest proposal: Displacement activities as an indicator of emotions in primates. Anim. Behav..

[16] Chelluri G.I., Ross S.R., Wagner K.E. (2013). Behavioral correlates and welfare implications of informal interactions between caretakers and zoo-housed chimpanzees and gorillas. Appl. Anim. Behav. Sci..

[17] Pomerantz O., Terkel J. (2009). Effects of positive reinforcement training techniques on the psychological welfare of zoo-housed chimpanzees (*Pan troglodytes*). Am. J. Primatol..

[18] Herrelko E.S., Vick S.-J., Buchanan-Smith H.M. (2012). Cognitive research in zoo-housed chimpanzees: influence of personality and impact on welfare. Am. J. Primatol..

[19] Ward S.J., Melfi V. (2013). The implications of husbandry training on zoo animal response rates. Appl. Anim. Behav. Sci..

[20] Savastano G., Hanson A., McCann C. (2003). The development of an operant conditioning training program for New World primates at the Bronx Zoo. J. Appl. Anim. Welf. Sci..

[21] Mitsui S., Yamamoto M., Nagasawa M., Mogi K., Kikusui T., Ohtani N., Ohta M. (2011). Urinary oxytocin as a noninvasive biomarker of positive emotion in dogs. Horm. Behav..

[22] Odendaal J.S.J., Meintjes R.A. (2003). Neurophysiological correlates of affiliative behaviour between humans and dogs. Vet. J..

[23] Handlin L., Hydbring-Sandberg E., Nilsson A., Ejdebäck M., Jansson A., Uvnäs-Moberg K. (2011). Short-term interaction between dogs and their owners: Effects on oxytocin, cortisol, insulin and heart rate—An exploratory study. Anthrozoös.

[24] Petersson M., Uvnäs-Moberg K., Nilsson A., Gustafson L.-L., Hydbring-Sandberg E., Handlin L. (2017). Oxytocin and cortisol levels in dog owners and their dogs are associated with behavioral patterns: An exploratory study. Front. Psychol..

[25] De Silva Vasconcellos A., Virányi Z., Range F., Ades C., Scheidegger J.K., Möstl E., Kotrschal K. (2016). Training reduces stress in human-socialised wolves to the same degree as in dogs. PLoS ONE.

[26] O’Brien J.K., Heffernan S., Thomson P.C., McGreevy P.D. (2008). Effect of positive reinforcement training on physiological and behavioural stress responses in the hamadryas baboon (*Papio hamadryas*). Anim. Welf..

[27] Behringer V., Stevens J.M.G., Hohmann G., Möstl E., Selzer D., Deschner T. (2014). Testing the effect of medical positive reinforcement training on salivary cortisol levels in bonobos and orangutans. PLoS ONE.

[28] Crockford C., Deschner T., Ziegler T., Wittig R. (2014). Endogenous peripheral oxytocin measures can give insight into the dynamics of social relationships: A review. Front. Behav. Neurosci..

[29] MacLean E.L., Gesquiere L.R., Gee N.R., Levy K., Martin W.L., Carter C.S. (2017). Effects of affiliative human–animal interaction on dog salivary and plasma oxytocin and vasopressin. Front. Psychol..

[30] Nagasawa M., Mitsui S., En S., Ohtani N., Ohta M., Sakuma Y., Onaka T., Mogi K., Kikusui T. (2015). Oxytocin-gaze positive loop and the coevolution of human-dog bonds. Science.

[31] Miller S.C., Kennedy C.C., DeVoe D.C., Hickey M., Nelson T., Kogan L. (2009). An examination of changes in oxytocin levels in men and women before and after interaction with a bonded dog. Anthrozoös.

[32] Powell L., Edwards K.M., Bauman A., Guastella A.J., Drayton B., Stamatakis E., McGreevy P. (2019). Canine endogenous oxytocin responses to dog-walking and affiliative human–dog interactions. Animals.

[33] Rault J.-L., van den Munkhof M., Buisman-Pijlman F.T.A. (2017). Oxytocin as an indicator of psychological and social well-being in domesticated animals: A critical review. Front. Psychol..

[34] Leeds A., Dennis P.M., Lukas K.E., Stoinski T.S., Willis M.A., Schook M.W. (2018). Validating the use of a commercial enzyme immunoassay to measure oxytocin in unextracted urine and saliva of the western lowland gorilla (*Gorilla gorilla gorilla*). Primates.

[35] Leeds C.A. (2019). Physiological Evaluation of Social Bonding in Western Lowland Gorillas (Gorilla gorilla gorilla). Ph.D. Thesis.

[36] Ioannou S., Gallese V., Merla A. (2014). Thermal infrared imaging in psychophysiology: Potentialities and limits. Psychophysiology.

[37] Stewart M., Webster J.R., Schaefer A.L., Cook N.J., Scott S.L. (2005). Infrared thermography as a non-invasive tool to study animal welfare. Anim. Welf..

[38] Travain T., Colombo E.S., Heinzl E., Bellucci D., Prato Previde E., Valsecchi P. (2015). Hot dogs: Thermography in the assessment of stress in dogs (*Canis familiaris*)—A pilot study. J. Vet. Behav..

[39] Dai F., Cogi N.H., Heinzl E.U.L., Dalla Costa E., Canali E., Minero M. (2015). Validation of a fear test in sport horses using infrared thermography. J. Vet. Behav. Clin. Appl. Res..

[40] Kuraoka K., Nakamura K. (2011). The use of nasal skin temperature measurements in studying emotion in macaque monkeys. Physiol. Behav..

[41] Nakayama K., Goto S., Kuraoka K., Nakamura K. (2005). Decrease in nasal temperature of rhesus monkeys (*Macaca mulatta*) in negative emotional state. Physiol. Behav..

[42] Kano F., Hirata S., Deschner T., Behringer V., Call J. (2016). Nasal temperature drop in response to a playback of conspecific fights in chimpanzees: A thermo-imaging study. Physiol. Behav..

[43] Grandi L.C., Heinzl E. (2016). Data on thermal infrared imaging in laboratory non-human primates: Pleasant touch determines an increase in nasal skin temperature without affecting that of the eye lachrymal sites. Data Brief.

[44] Cronbach L.J., Meehl P.E. (1955). Construct validity in psychological tests. Psychol. Bull..

[45] Vonk J., Jett S. (2018). “Bear-ly” learning: Limits of abstraction in black bear cognition. Anim. Behav. Cognit..

[46] Friard O., Gamba M. (2016). BORIS: A free, versatile open-source event-logging software for video/audio coding and live observations. Methods Ecol. Evol..

[47] Altmann J. (1974). Observational study of behavior: Sampling methods. Behaviour.

[48] Plowman A.B. (2008). BIAZA statistics guidelines: Toward a common application of statistical tests for zoo research. Zoo Biol..

[49] Sato Y., Hirata S., Kano F. (2019). Spontaneous attention and psycho-physiological responses to others’ injury in chimpanzees. Anim. Cognit..

[50] Proctor H.S., Carder G. (2015). Nasal temperatures in dairy cows are influenced by positive emotional state. Physiol. Behav..

[51] Kosonogov V., De Zorzi L., Honoré J., Martínez-Velázquez E.S., Nandrino J.-L., Martinez-Selva J.M., Sequeira H. (2017). Facial thermal variations: A new marker of emotional arousal. PLoS ONE.

[52] Nakanishi R., Imai-Matsumura K. (2008). Facial skin temperature decreases in infants with joyful expression. Infant Behav. Dev..

[53] Salazar-Lopez E., Dominguez E., Ramos V.J., de la Fuente J., Meins A., Iborra O., Galvez G., Rodriguez-Artacho M.A., Gomez-Milan E. (2015). The mental and subjective skin: Emotion, empathy, feelings and thermography. Conscious. Cogn..

[54] Singh J., Kumar S., Arora A.S. (2018). Thermographic evaluation of mindfulness meditation using dynamic IR imaging. Infrared Phys. Technol..

[55] Chotard H., Ioannou S., Davila-Ross M. (2018). Infrared thermal imaging: Positive and negative emotions modify the skin temperatures of monkey and ape faces. Am. J. Primatol..

[56] Ioannou S., Chotard H., Davila-Ross M. (2015). No strings attached: Physiological monitoring of rhesus monkeys (*Macaca mulatta*) with thermal imaging. Front. Behav. Neurosci..

[57] Patel F., Whitehouse-Tedd K., Ward S.J. (2019). Redefining human–animal relationships: An evaluation of methods to allow their empirical measurement in zoos. Anim. Welf..

[58] Wagner K.E., Hopper L.M., Ross S.R. (2016). Asymmetries in the production of self-directed behavior by chimpanzees and gorillas during a computerized cognitive test. Primates.

[59] Yamanashi Y., Matsuzawa T. (2010). Emotional consequences when chimpanzees (*Pan troglodytes*) face challenges: Individual differences in self-directed behaviours during cognitive tasks. Anim. Welf..

[60] Ross S.R., Wagner K.E., Schapiro S.J., Hau J. (2010). Ape behavior in two alternating environments: Comparing exhibit and short-term holding areas. Am. J. Primatol..

[61] Salmi R. (2015). Vocal Communication of Wild Western Gorillas (*Gorilla gorilla*). Ph.D. Thesis.

[62] Salmi R., Hammerschmidt K., Doran-Sheehy D.M. (2013). Western gorilla vocal repertoire and contextual use of vocalizations. Ethology.

[63] Spiezio C., Vaglio S., Scala C., Regaiolli B. (2017). Does positive reinforcement training affect the behaviour and welfare of zoo animals? The case of the ring-tailed lemur (*Lemur catta*). Appl. Anim. Behav. Sci..

[64] Hinde K., Muth C., Maninger N., Ragen B.J., Larke R.H., Jarcho M.R., Mendoza S.P., Mason W.A., Ferrer E., Cherry S.R. (2016). Challenges to the pair bond: Neural and hormonal effects of separation and reunion in a monogamous primate. Front. Behav. Neurosci..

[65] Snowdon C.T., Pieper B.A., Boe C.Y., Cronin K.A., Kurian A.V., Ziegler T.E. (2010). Variation in oxytocin is related to variation in affiliative behavior in monogamous, pairbonded tamarins. Horm. Behav..

[66] Kuhar C.W., Bettinger T.L., Laudenslager M.L. (2005). Salivary cortisol and behaviour in an all-male group of western lowland gorillas (*Gorilla g. gorilla*). Anim. Welf..

[67] Heintz M.R., Santymire R.M., Parr L.A., Lonsdorf E.V. (2011). Validation of a cortisol enzyme immunoassay and characterization of salivary cortisol circadian rhythm in chimpanzees (*Pan troglodytes*). Am. J. Primatol..

[68] Cross N., Pines M.K., Rogers L.J. (2004). Saliva sampling to assess cortisol levels in unrestrained common marmosets and the effect of behavioral stress. Am. J. Primatol..

[69] Talge N.M., Donzella B., Kryzer E.M., Gierens A., Gunnar M.R. (2005). It’s not that bad: Error introduced by oral stimulants in salivary cortisol research. Dev. Psychobiol..

[70] Toda M., Morimoto K., Nagasawa S., Kitamura K. (2004). Effect of snack eating on sensitive salivary stress markers cortisol and chromogranin A. Environ. Health Prev. Med..

[71] Gröschl M., Wagner R., Rauh M., Dörr H.G. (2001). Stability of salivary steroids: The influences of storage, food and dental care. Steroids.

[72] Lutz C.K., Tiefenbacher S., Jorgensen M.J., Meyer J.S., Novak M.A. (2000). Techniques for collecting saliva from awake, unrestrained, adult monkeys for cortisol assay. Am. J. Primatol..

[73] Fagot J., Gullstrand J., Kemp C., Defilles C., Mekaouche M. (2014). Effects of freely accessible computerized test systems on the spontaneous behaviors and stress level of Guinea baboons (*Papio papio*). Am. J. Primatol..

[74] Elder C.M. (2001). Dissociation of cortisol and behavioral indicators of stress in an orangutan (*Pongo pygmaeus*) during a computerized task. Primates.

[75] Novak M.A., Hamel A.F., Kelly B.J., Dettmer A.M., Meyer J.S. (2013). Stress, the HPA axis, and nonhuman primate well-being: A review. Appl. Anim. Behav. Sci..

[76] Wielebnowski N. (2003). Stress and distress: Evaluating their impact for the well-being of zoo animals. J. Am. Vet. Med. Assoc..

[77] Engert V., Merla A., Grant J.A., Cardone D., Tusche A., Singer T. (2014). Exploring the use of thermal infrared imaging in human stress research. PLoS ONE.

[78] Bosch J.A., Veerman E.C.I., de Geus E.J., Proctor G.B. (2011). α-Amylase as a reliable and convenient measure of sympathetic activity: Don’t start salivating just yet!. Psychoneuroendocrinology.

[79] Anacker A., Beery A. (2013). Life in groups: The roles of oxytocin in mammalian sociality. Front. Behav. Neurosci..

[80] Spinka M. (2019). Animal agency, animal awareness and animal welfare. Anim. Welf..

[81] Melfi V. (2013). Is training zoo animals enriching?. Appl. Anim. Behav. Sci..

